# In Situ Anodic Transition and Cathodic Contamination Affect the Overall Voltage of Alkaline Water Electrolysis

**DOI:** 10.3390/molecules29225298

**Published:** 2024-11-09

**Authors:** Zheng Liu, Zhaoyu Liu, Lingxing Zan, Yu Sun, Huizhen Han, Zhe Li, Han Wang, Ting Cao, Yao Zhu, Haiyang Lv, Yuxuan Liu, Juzhe Liu, Xin Bo

**Affiliations:** 1 SEPA Key Laboratory of Eco-Industry, Chinese Research Academy of Environmental Sciences, Beijing 100012, China; 2 Key Laboratory of Applied Surface and Colloid Chemistry, Ministry of Education, Institute of New Concept Sensors and Molecular Materials, School of Chemistry and Chemical Engineering, Shaanxi Normal University, Xi’an 710119, China; 3 Key Laboratory of Chemical Reaction Engineering of Shaanxi Province, College of Chemistry & Chemical Engineering, Yan’an University, Yan’an 716000, China; 4 Key Laboratory of Resources and Environmental Systems Optimization, Ministry of Education, College of Environmental Science and Engineering, North China Electric Power University, Beijing 102206, China

**Keywords:** NiFe (oxy)hydroxide, oxygen evolution reaction, hydrogen evolution reaction, aging, alkaline water electrolysis

## Abstract

NiFe (oxy)hydroxide has been widely used as a benchmark anodic catalyst for oxygen evolution reactions (OERs) in alkaline water electrolysis devices; however, the energy saving actually takes contributions from both the anodic OER and cathodic hydrogen evolution reaction (HER). In this work, we observe the catalytic promotion upon the in situ-derived NiFe (oxy)hydroxide from the NiFe alloy monolithic electrode and also point out that the coupled nickel cathode is contaminated, leading to the loss of HER activity and a reduction in overall efficiency. It is found that Ni^2+^ and Fe^3+^ cations are inevitably detached from the anode into the electrolyte and electrodeposited on the nickel cathode after the three-month industrial simulation. This research presents the significant enhancement of the oxygen evolution catalysis using an in situ aging process and emphasizes that the catalytic application should not only be isolated on the half reaction, but a reasonable coupled electrode match to get rid of the contamination from the electrolyte is also of great significance to sufficiently present the intrinsic catalytic yielding for the real application.

## 1. Introduction

Alkaline water electrolysis (AWE) is one of the most efficient approaches to obtain green hydrogen fuel [1,2,3,4,5]. However, there is still room to further reduce the extra energy input for water-splitting processes by using advanced catalytic electrodes [6,7,8,9,10]. For the real industry, pure nickel mesh and/or nickel foam are used as a commercial bi-functional electrode in both cathodic and anodic chambers for flowing-electrolyzer–stacks (FESs) in alkaline media (e.g., KOH aq.), in which the sluggish four-electron transferring on the anodic oxygen evolution reaction (OER) is regarded as the critical shortboard [6,9]. To this regard, NiFe (oxy)hydroxide was developed and found to be a benchmark catalyst for OERs, improving the electrocatalytic efficiency by 200~300 mV onset potential drop [10,11]. To further augment the OER activity, strategic doping of elements, including Cr, Mn, Ce, Mo, V, W, etc., was conducted within the NiFe (oxy)hydroxide electrode [12,13,14,15,16,17,18,19,20,21,22,23,24,25,26].

**Figure 1:**
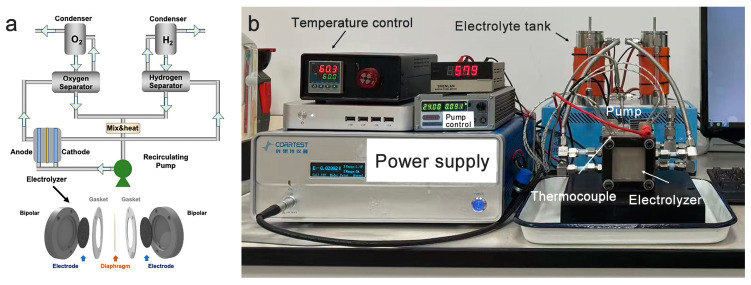
(**a**) Scheme of AWE system and electrolyzer; (**b**) photo of lab-built AWE system.

As the advanced catalysts applied into the industrial FES (Figure 1), the KOH electrolyte is pumped into cathodic and anodic chambers at the same time [27,28]. The generated hydrogen and oxygen with the electrolyte experience gas/liquid separation and condenser units so that the circulated electrolyte is re-fed into the electrolyzer. Although there is a diaphragm preventing gas and electrolyte contacting directly to the counter chamber, the external recycling system permits the electrolyte converging for dynamic pH equilibrium. The electrolytes in cathodic and anodic are not independent. Therefore, some issues occurred during the application of these catalytic materials in industrial alkaline water electrolysis. (1) Elements such as Fe, Cr, Mn, Ni, Mo, etc., in the anode and the stainless steel pipeline can be partially dissolved into the strong alkaline media during OER processes [13,29,30,31]; (2) the nomadic metal ions in the electrolyte broadcast and electrodeposit to contaminate the cathode following the circulation route even with the existence of a diaphragm [32,33]; (3) the consistent degradation on the anode and contamination on the cathode lead to material depletion and deactivation, respectively. For example, the loss of Fe in the NiFe oxyhydroxide anode can even change the OER kinetics from LOM to AEM, slowing down the reaction rate [34]. (4) Moreover, the mechanical failure, such as bubble attack, stress, morphologic change, etc., can also trigger the electrode destruction [34].

In order to investigate the above issues, we introduced a series of NiFe alloy foams as the anodes, which can provide abundant Ni and/or Fe species from the bulky frames to observe the resultant influence during the industrial AWE simulation; the in situ phase transition from the surface NiFe alloy into the NiFe (oxy)hydroxide presented a significant improvement on OER performance [35], while the long-term simulation of the industrial water electrolysis scenario also disclosed that the nickel cathode was severely coated with a layer of Ni/Fe composite. The loose and dendric crystalline on the cathode was fragile and less-catalytic for HER. As a result, although the optimal NiFe alloy yields an outstanding NiFe (oxy)hydroxide active phase for the OER, the de-activated cathode for HER reduces the overall efficiency improvement. From this aspect, it is emphasized that the industrial AWE application should consider not only the active electrode but also the contamination from the undesired impurities.

## 2. Results and Discussion

As the scheme shows in Figure 1, we simulated the industrial AWE by using various NiFe alloy foams (as well as pure Ni and Fe foams) as anodes and pure nickel foam as cathodes. The photos of the pristine nickel foam, nickel–iron alloy foams, and iron foams before and after AWE aging are displayed in Figures S1 and S2. The pristine foams containing nickel present a silver color, while the iron foam is gray. After utilization in the electrolysis cell for three months, all the metallic foams turned black (Figure S2). X-ray diffraction (XRD) patterns of pristine nickel foam, nickel–iron alloy foams, and iron foams before the electrochemical aging process are displayed in Figures S3 and S4. The peaks at 44.5°, 51.8°, and 76.3° correspond to the identical peaks of nickel (111), (200), and (220) facets. Meanwhile, the XRD patterns of iron foam present two packs of signals ascribed to Fe (PDF#06-0696) and FeO (PDF#06-0615). The peaks of nickel–iron alloy foams are close to the typical peaks of nickel with a shift to lower angle degree, indicating a substitutional solid state of nickel–iron alloy. After aging in the electrolysis cell, only metal signals are detected on aged Ni and NiFe anodes (Figures S3 and S5), which may be ascribed to the amorphous structure and relatively low mass loading of the aged layer (Figures S3 and S5), while the aged Fe anode presents the identical iron (oxy)hydroxide peaks (Figure S5, PDF#19-0629).

**Figure 2:**
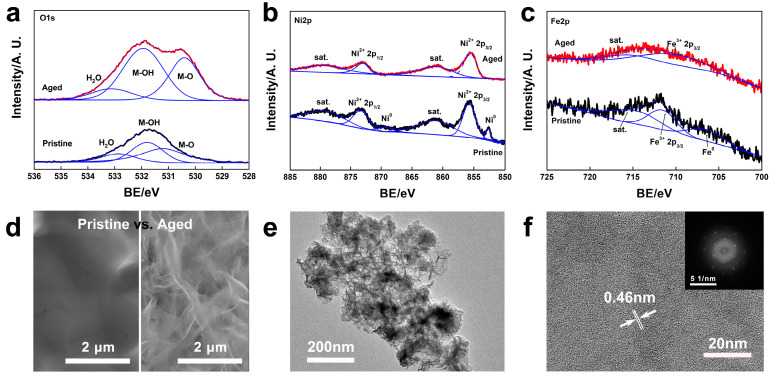
XPS data of O1s (**a**), Ni2p (**b**), and Fe2p (**c**) from Ni3Fe foam before and after alkaline water electrolysis aging; SEM photos of Ni_3_Fe foam before and after aging (**d**); TEM (**e**) and HR-TEM (**f**) of the active layer peeled from Ni_3_Fe foam after alkaline water electrolysis aging (inset, FFT pattern from HR-TEM).

X-ray photoelectron spectroscopic (XPS) data of the corresponding Ni, Fe, and O on the typical Ni_3_Fe foam before and after electrolysis aging are also demonstrated in Figure 2a–c. After electrolysis aging, M-O and M-OH bonds significantly increased (Figure 2a), indicating the surficial atom reconstruction to the active (oxy)hydroxide during water oxidation processes. Meanwhile, the metallic state signals ascribed to Ni^0^ and Fe^0^ also disappeared, indicating that the surficial Ni^0^ and Fe^0^ were transformed to Ni^2+^ and Fe^3+^ states, respectively. Notably, the aged-Fe signal is much weaker than that of the pristine anode, also suggesting Fe’s degradation. As a comparison, the XPS data of Ni and Fe foams before and after electrolysis aging are shown in Figures S6–S9. Similarly, the pristine state of the metallic Ni^0^ was aged into Ni^2+^, while only Fe^3+^ species were found on the surface of Fe foam before and after aging, indicating that the surface of Fe foam is always oxidized.

The corresponding scanning electron microscopic (SEM) photos in Figure 2d and Figures S10–S13 display the morphologic changes in the metallic/alloy foams surfaces. The pristine foams present network frames with smooth surfaces, while the aged metallic/alloy foams demonstrate rough wrinkles on the surface, which is a typical (oxy)hydroxide structure [11]. Particularly, the activated surface layer was carefully peeled off from the Ni_3_Fe foam by ultrasonication, and the corresponding transmission electron microscopic (TEM) photo is displayed in Figure 2e,f. The lattice fringe is around 0.46 nm, representing (002) facets of the NiOOH intermediate (59-0464, 2023 International Centre for Diffraction Data).

**Figure 3:**
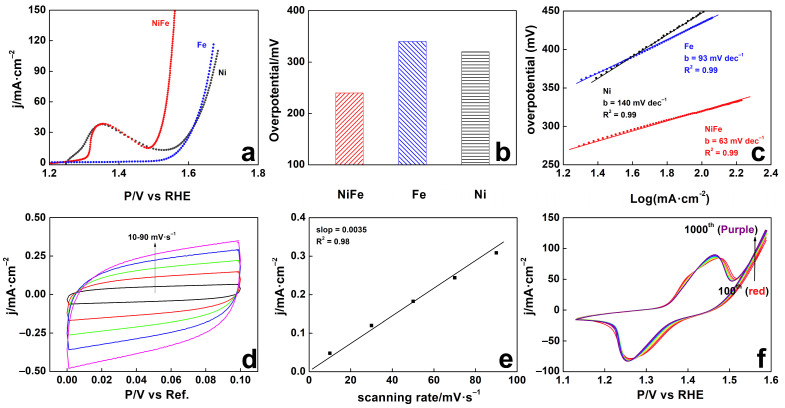
OER performances were collected in 1 M KOH electrolyte; LSVs (**a**) of Ni_3_Fe, Fe, and Ni foams before and after alkaline water electrolysis aging with 80% iR compensation and the corresponding overpotential (**b**) at 10 mA·cm^−2^ and derived Tafel slope values (**c**); CVs under various scanning rates (black 10 mV s^−1^, red 30 mV s^−1^, green 50 mV s^−1^, blue 70 mV s^−1^, magenta 90 mV s^−1^) for ECSA evaluation (**d**,**e**); (**f**) 1000 CVs of the aged Ni_3_Fe anode without iR compensation at the scanning ramp of 50 mV·s^−1^.

The OER performance after aging in the electrolysis cell is displayed in Figure 3a and Figures S14–S16. In Figure S14, the pristine Ni_3_Fe demonstrates relatively higher OER activity, while the activity on Fe foam is relatively low. After aging in the electrolysis (Figure S15), the OER performance of the Ni_3_Fe foam is essentially increased, and the corresponding onset potential is only around 1.47 V vs. RHE. Compared with the aged Ni and Fe foams (Figure 3a), the onset potential is reduced by nearly 150 mV. It is also noted that the OER performance of Ni and Fe foams are actually decreased after electrolysis aging (Figure S16), indicating that the aging-activation process only works on NiFe alloy foams. The elemental ratio of Ni_3_Fe foam is also consistent with the reported studies that Ni:Fe = 3:1 is optimal for OER process [29]. The corresponding overpotential values under the yielding current density of 100 mA·cm^−2^ of the aged anodes are read in Figure 3b, showing that the feedback overpotential of the aged Ni_3_Fe foam is only around 310 mV compared with 420 mV and 450 mV from Fe and Ni foams, respectively. The derived Tafel slopes (Figure 3c) from Figure 3a also disclose the smallest value on aged Ni_3_Fe foam, indicating that the rate-determining step (RDS) is M-OH + OH^−^ → M-O + H_2_O + e^−^ and/or M + OH^−^ → M-OH + e^−^ [15,36]. As the micro-structure shows, the wrinkled surface on the aged electrode consequently leads to an enlarged electrochemical surface area (ECSA). Therefore, the ECSAs of Ni_3_Fe foams before and after aging are also evaluated by measuring the cyclic voltammetries (CVs) under various scanning rates in non-Faradaic windows (Figure 3d,e and Figure S18). The electrochemical active surface area is obviously enlarged after electrolysis aging on Ni_3_Fe foam. Similarly, other ECSAs of the metallic and alloy foams are also enlarged after aging (Figures S17–S21). Lastly, the CVs (Figure 3f) of the Ni_3_Fe electrode with 1000 recycles also reflect the gradual enhancement of the OER activity.

In summary, when the Ni_3_Fe alloy foam was used as the electrode in the AWE system, the surface of the anode can in situ transform to the active, wrinkled NiFe (oxy)hydroxide species with an enlarged surface area and can output the enhanced OER activity.

**Figure 4:**
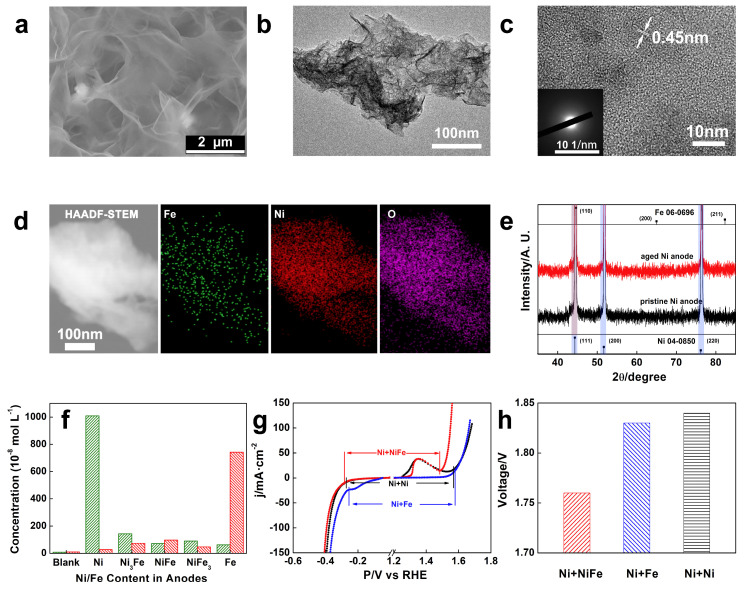
SEM (**a**) of Ni foam cathode coupled with Ni_3_Fe foam anode and after electrolysis aging; TEM (**b**) and HR-TEM (**c**) of the contaminated layer peeled from the Ni foam cathode coupled with Ni_3_Fe foam anode and after electrolysis aging (inset, SAED); (**d**) TEM-EDS mapping of Fe, Ni and O in the selected area; (**e**) XRD patterns of the Ni foam cathode before and after electrolysis aging; (**f**) ICP-OES of the Ni and Fe ion contents in the KOH electrolyte (green for Ni and red for Fe); (**g**) LSVs of the overall electrochemical performance of water electrolysis from the Ni||Ni, Ni||Ni_3_Fe, and Ni||Fe groups and their corresponding overall AWE voltage (**h**).

Although there is an obvious OER improvement on the aged Ni_3_Fe foam, we should not isolate the catalytic contribution from both the anode and cathode to overall water electrolysis. The changes in the electrolyte and the cathodes are also discussed. In Figure S22, the pure nickel foams coupled with various foams (denoted as Ni||Ni, Ni||NiFe_3_, Ni||NiFe, Ni||Ni_3_Fe, and Ni||Fe) all turn into gray and black as the Fe content increases. Particularly, the couplet group of Ni||Fe gives the most severely qualitative change that obvious dendric coatings are found on the substrate. The micro-structural changes were further analyzed with the SEM in Figure 4a and Figures S23–S27. A TEM image of a specific aged cathode with Ni_3_Fe counter is displayed in Figure 4b, depicting a wrinkled 2D structure. The HR-TEM (Figure 4c) confirms the existence of the NiOOH phase by observation of the lattice fringe of 0.45 nm (002). Notably, the formed layer mostly has amorphous structure. The EDS mapping (Figure 4d) on the selected area proves that the foreign Fe dopes into the amorphous NiOOH layer. The chemical equation can be described as (1) 2H_2_O + 2e^−^ ⇌ H_2_ + 2OH^−^ and (2) M^m+^ + N^n+^ + (m + n)OH^−^ ⇌ MN(OH)_m+n_ (M and N represent metallic cations) [24]. In a word, the contaminated layer of the nickel cathode (e.g., coupled with Ni_3_Fe foam) is supposed to be NiFe (oxy)hydroxide composite. Consequently, the relevant ECSAs of the aged cathodes should also be enlarged (Figures S28–S33). The severe Fe coating in the Ni||Fe group results in a massive dendric–crystalline deposition on the aged Ni cathode, yielding the largest ECSA change (Figure S33). Notably, the XRD patterns (Figure 4e and Figure S34) of the aged cathodes only show the peaks of the metallic substrate beneath, indicating the thin amorphous-like layer.

Generally speaking, the metal (oxy)hydroxide should be chemically stable in strong alkaline media. According to K_sp_ values of Ni(OH)_2_ and Fe(OH)_3_, the solubility values of Ni^2+^ and Fe^3+^ in 1 M KOH media are only 2 × 10^−15^ and 4 × 10^−38^ M, respectively, which are much lower than those of the measured concentration in Figure 4f [37]. After AWE, the nickel cation content significantly increases (~10^−5^ M) with the use of pure Ni anode. When we used NiFe foams as anodes, nickel and iron cations in the electrolyte after long-term electrolysis aging are also improved (Ni^2+^: 6~8 × 10^−7^, Fe^3+^: 4~10 × 10^−7^), leading to the contamination as well. Obviously, the pure Fe anode is more feasible to be corroded in alkaline so that the coupled cathode is heavily coated, and the Fe foam is not recommended as the anode. This observation also highlights that it applying some Fe-rich materials as the anode in AWE should be paid more attention to [38].

The real content of both Ni^2+^ and Fe^3+^ are essentially higher than the theoretical solubility of hydroxide since the anode is charged under the oxidized potential. Then, the dissolved metallic cations form the (hydroxo) complexes so that there is no precipitation in the electrolyte and the supersaturated cationic contents are obtained. For Fe^3+^, a series of Fe^3+^ hydroxo complexes, ranging from [Fe(OH)_4_]^−^, [Fe(OH)_5_]^2−^, [Fe(OH)_6_]^3−^, etc., can be formed in the strong alkaline media [39,40,41]. When it comes to Ni^2+^, the case seems different since there is no typical nickel hydroxo complex, but it can be coordinated with H_2_O, NH_3_, etc. [42]. Alternatively, the Ni(OH)_2_ (s) transforms to a nickelate species in alkaline media, followed by the reaction of Ni(OH)_2_ + OH^−^ ⇌ HNiO_2_^−^ + H_2_O, giving a significant increase in K_sp_ to 10^−5^ [43,44]. Therefore, the metal cations are gradually concentrated in the electrolyte, and the cathode contamination process can be described as M^m+^ + N^n+^ + (m + n)OH^−^ → MN(OH)_(m+n)_ above, where the cations are released from the nomadic complexes in the electrolyte [24]. Notably, with the reduction in Fe^3+^, the reaction on the aged cathode of the Ni||Fe group should be Fe^3+^ + 3e → Fe^0^.

Consequently, the HER performances (Figures S35–S37) of the aged-nickel cathodes are also influenced by the cationic contamination. To be specific, the HER performance of the Ni||Ni group is nearly as unchanged as the same Ni coatings; however, the output of the Ni||Ni_3_Fe group is as decreased as the foreign (oxy)hydroxide coatings. Although there is an increase in the HER activity of the Ni||Fe group, which may result from the enlarged ECSA, the coated Fe impurities on the cathode are very loose and fragile, taking the high risk of slag detachment upon the weak binding and short-circuit to burn the electrolyzer. As a result, since the degradation on contaminated nickel cathodes, the improvement of the overall water-splitting efficiency is actually discounted, as shown in Figure 4g,h. Moreover, in real industrial application scenarios, there are many other factors influencing the electrolyzer performance, such as the resistance of electrolyte and diaphragm, electrolyte-flowing and gas bubble effect, etc. Therefore, the development of the advanced catalytic electrode should not only focus on its activity independently, the match of couplet counter electrode is also significant for practical utilization.

## 3. Materials and Methods

Materials preparation: All the chemicals were purchased from a supplier and directly used without further purification. The nickel foam (1 mm thickness, 110 ppm), nickel–iron alloy foam (1 mm thickness, 110 ppm), and iron plate (1 mm thickness) were customized from JSD (Suzhou, China) supplier. Before use, the metallic foams were firstly sonicated in diluted hydrochloric acid (~4 M) solution for 10 min to remove the oxides on the surface and then washed with Milli-Q water (18.2 MΩ·cm, Millipore, Burlington, MA, USA), acetone, and ethanol. The metallic foams were then cut into a certain size of 3 × 5 cm^2^ and utilized into an electrochemical cell filled with 1 M KOH electrolyte (Sigma, St. Louis, MO, USA, electronic degree, >99.999%). To be specific, nickel foams were used as cathodes, while the counter anodes were arranged as nickel foams, nickel–iron foams (Ni:Fe ratios = 3:1, 1:1, and 1:3), or iron foam. The five cells were then applied under a constant electrolysis current density of 300 mA·cm^−2^ (DC power supply, HYELEC, HY3010B, Hangzhou, China) for three months and fed Milli-Q water to maintain the electrolysis. The electrodes and electrolyte were thereafter dissembled from the electrolysis cell and collected for analysis, respectively.

Materials characterizations: Scanning electron microscopy (SEM) was carried out with Quanta 200F (FEI, Hillsboro, OR, USA) and S5500 (HITACHI, Tokyo, Japan), with the acceleration voltages of 20 kV and 30 kV, respectively. X-ray diffraction (XRD) patterns were obtained using a Rigaku Smartlab diffractometer (Akishima, Japan, 40 kV, 200 mA) with Cu Kα radiation at the scanning rate of 5°·min^−1^ from 30 to 90 2θ-degree. X-ray photoelectron spectroscopy (XPS) measurements were collected on Thermo ESCALAB 250Xi (Waltham, MA, USA) by using Al Kα X-ray as the excitation source, and the binding energies (BEs) were calibrated using contaminant C at 284.6 eV. High-resolution transmission electron microscope (HR-TEM) images were captured with HT7700 (HITACHI, 300 kV). The electrolyte was analyzed with Agilent 720 (Santa Clara, CA, USA) for ICP-OES (Inductively Coupled Plasma Optical Emission Spectrometer).

Electrochemical measurements: The obtained cathodes and anodes were thereafter used as working electrodes (exposed geographic area 1.0 × 1.0 cm^2^) in a standard three-electrode system, where a graphite rod and Hg/HgO (in saturated KCl) were fixed as counter and reference electrode, respectively. The linear sweep voltammetries (LSVs, scanning rate of 5 mV·s^−1^) and cyclic voltammetries (CVs) were collected using a CHI760E potentiostat (Bee Cave, TX, USA) in 1 M KOH electrolyte (pH = 13.7). The recorded potentials were converted into the value vs. reversible hydrogen electrode (RHE) by the following equations: E_RHE_ = E_msd_ + 0.098 + 0.059pH. Notably, iR correction was taken into consideration as well. The electrochemical surface areas (ECSAs) of the catalysts were estimated from the double-layer capacitance (C_dl_), measured via cyclic voltammetry at various scan rates in the non-Faradaic process region [45]. As given in the Equations j = *ν*·C_dl_ and ECSA ∝ C_dl_, j and *v* are the double-layer charging-current density and scan rate, respectively. The merit of the simulated C_dl_ represents the ECSA.

## 4. Conclusions

In conclusion, we initially utilized Ni, NiFe, and Fe foams as the monolithic anodes which can directly converse into the active Ni-, NiFe-, and/or Fe- (oxy)hydroxides from the bulky frame during AWE in alkaline media. The optimal Ni_3_Fe foam after electrolysis aging presents the typical NiFe (oxy)hydroxide species and performs an improved OER activity. However, the efficiency improvement on anode does not fully satisfy the overall water splitting. As the dissolution of the cation impurities from the anode, the contaminated counter cathodes hold back the total efficiency for water electrolysis. Although the design for advanced anodic OER catalysts is still important, the chemical stability for anodes in strong alkaline media under corrosive OER current and the anti-contamination tolerance for cathodes should also be taken into consideration. The realization of highly efficient water splitting is not an isolated object but a systematic interaction.

## Data Availability

Data are contained within the article and Supplementary Materials.

## Supplementary Materials

The following supporting information can be downloaded at: https://www.mdpi.com/article/10.3390/molecules29225298/s1. Figures S1 and S2, Photos the anodes before and after aging. Figures S3–S5, XRD patterns of the anodes before and after aging. Figures S6 and S7, XPS data of O1s and Ni2p on Ni anode before and after aging. Figures S8 and S9, XPS data of O1s and Fe2p on Fe anode before and after aging. Figures S10–S13, SEM of Ni, NiFe, NiFe_3_ and Fe anodes before and after aging. Figures S14–S16, OER performance of the anodes before and after aging. Figures S17–S21, ECSA information of anodes. Figure S22, Photos of Ni cathodes with various anodes before and after aging (wet). Figures S23–S27, SEM Ni cathodes with various anodes after aging. Figures S28–S33, ECSA information of the aged cathodes. Figure S34, XRD patterns of Ni cathodes before and after aging. Figures S35–S37, HER performance of the aged Ni cathodes.
