# Supplementary material for: In Situ Anodic Transition and Cathodic Contamination Affect the Overall Voltage of Alkaline Water Electrolysis
Source: Molecules. 2024 Nov 9;29(22):5298. doi: 10.3390/molecules29225298 (PMC11596874; doi:10.3390/molecules29225298)
Supplement: Supplementary file 1 [file molecules-29-05298-s001.zip › molecules-3241967-supplementary.pdf]

In Situ Anodic Transition and Cathodic Contamination Affect the Overall Voltage of Alkaline Water Electrolysis

Zheng Liu [1,†], Zhaoyu Liu [2,†], Lingxing Zan [3], Yu Sun [2], Huizhen Han [2], Zhe Li [3], Han Wang [1], Ting Cao [1], Yao Zhu [1], Haiyang Lv [1], Yuxuan Liu [4], Juzhe Liu [4,*] and Xin Bo [2,*]

[1] SEPA Key Laboratory of Eco-Industry, Chinese Research Academy of Environmental Sciences, Beijing 100012, China;

[2] Key Laboratory of Applied Surface and Colloid Chemistry, Ministry of Education, Institute of New Concept Sensors and Molecular Materials, School of Chemistry and Chemical Engineering, Shaanxi Normal University, Xi'an 710119, China;

[3] Key Laboratory of Chemical Reaction Engineering of Shaanxi Province, College of Chemistry & Chemical Engineering, Yan'an University, Yan'an 716000, China;

[4] Key Laboratory of Resources and Environmental Systems Optimization, Ministry of Education, College of Environmental Science and Engineering, North China Electric Power University, Beijing 102206, China

* Correspondence: liujuzhe@ncepu.edu.cn (J.L.); box@snnu.edu.cn (X.B.)

† These authors contribute equally to this work.

Figures and discussion

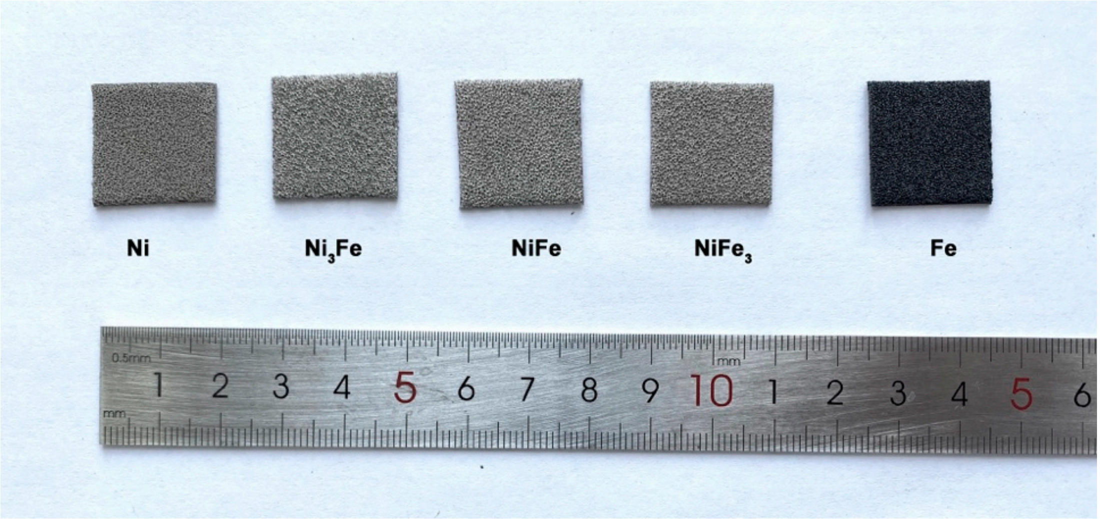

Figure S1 Photos of pristine Ni, NiFe and Fe foams before water electrolysis aging.

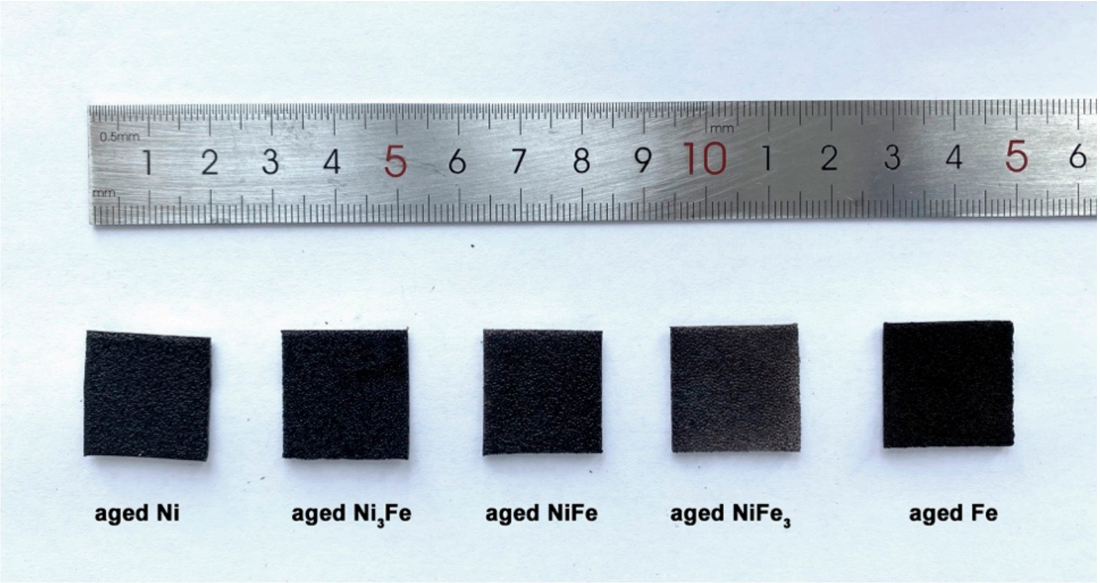

Figure S2 Photos of Ni, NiFe and Fe foams after water electrolysis aging.

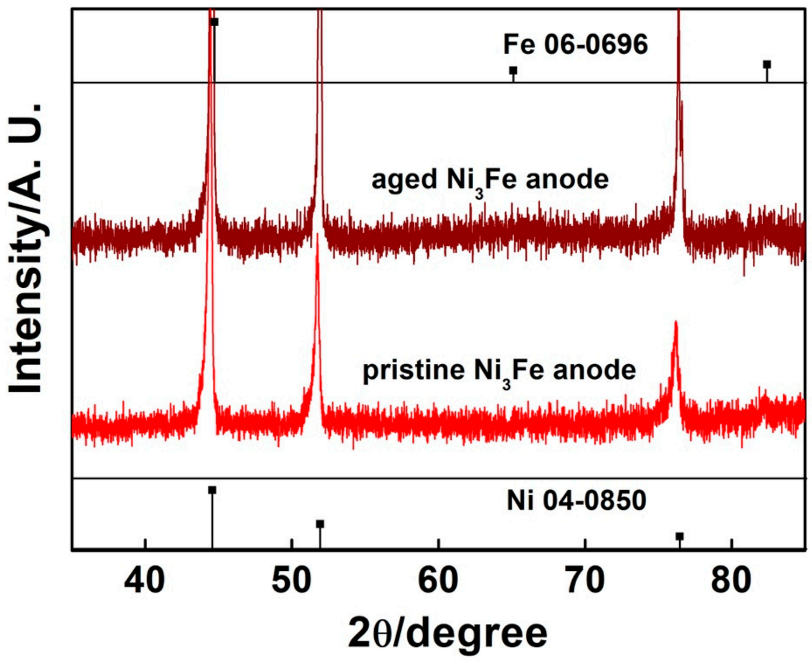

Figure S3 XRD patterns of Ni_3Fe foam before and after alkaline water electrolysis aging.

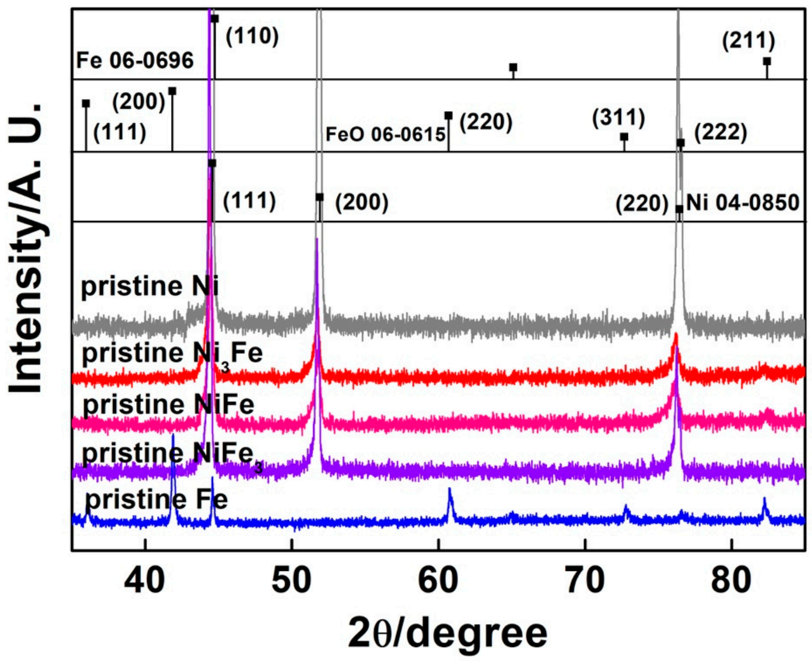

Figure S4 XRD patterns of Ni, NiFe and Fe foams before water electrolysis aging.

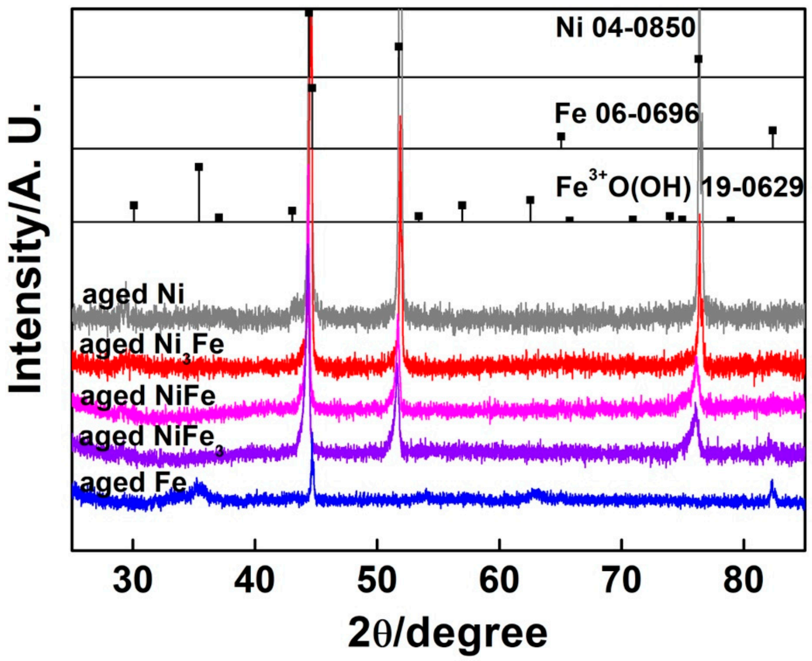

Figure S5 XRD patterns of Ni, NiFe and Fe foams after water electrolysis aging.

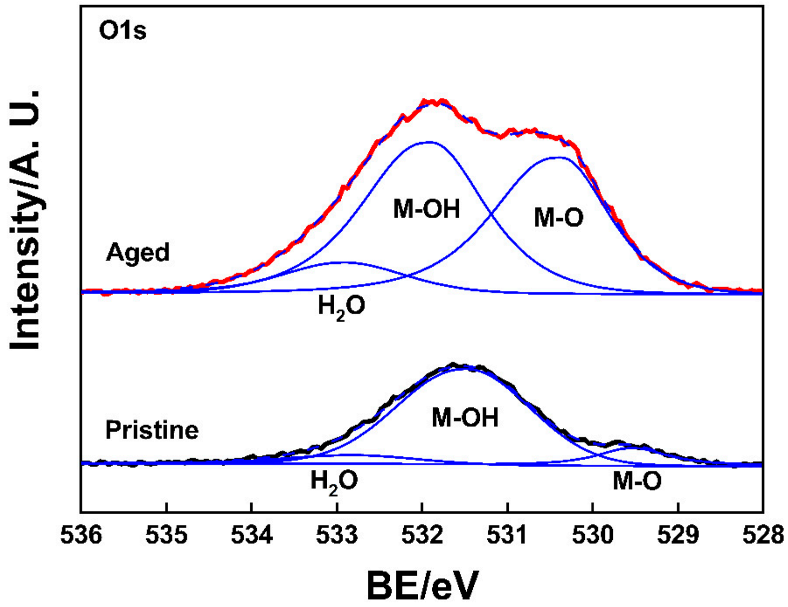

Figure S6 XPS data of O1s from anodic Ni foam before and after alkaline water electrolysis aging.

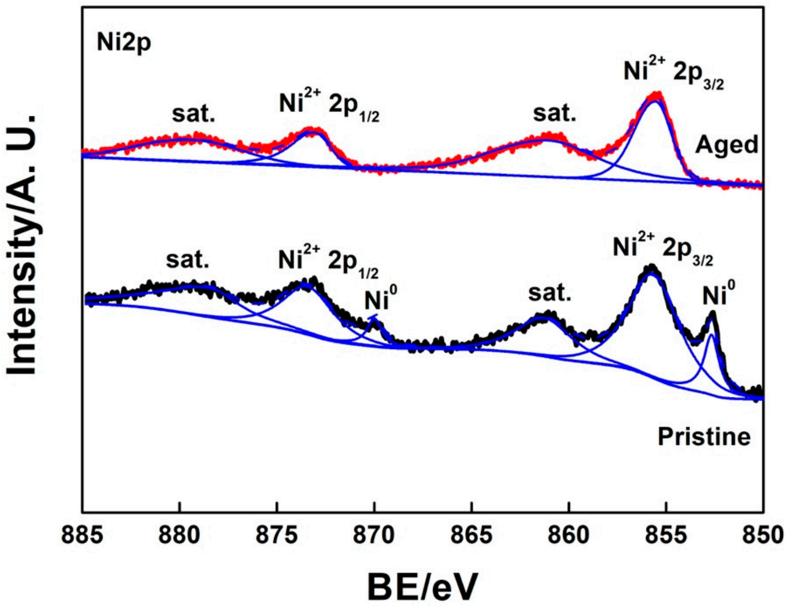

Figure S7 XPS data of Ni2p from anodic Ni foam before and after alkaline water electrolysis aging.

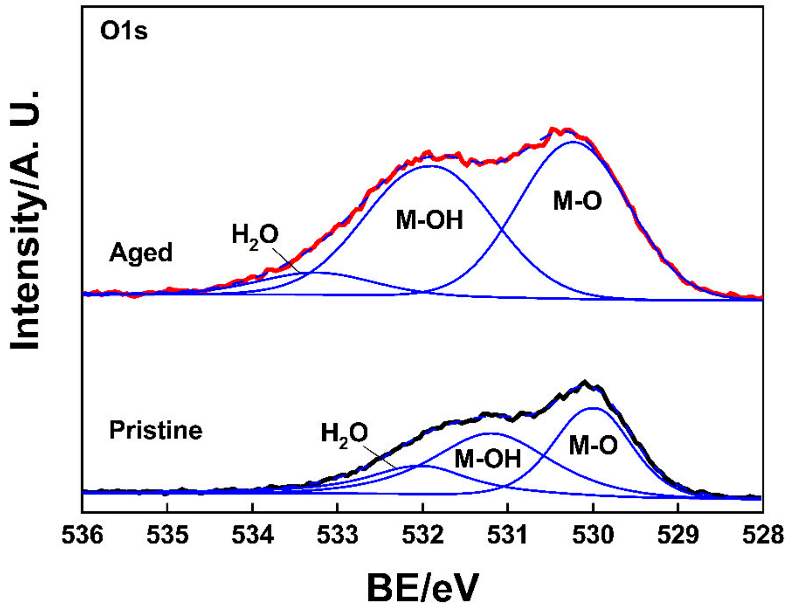

Figure S8 XPS data of O1s from anodic Fe foam before and after alkaline water electrolysis aging.

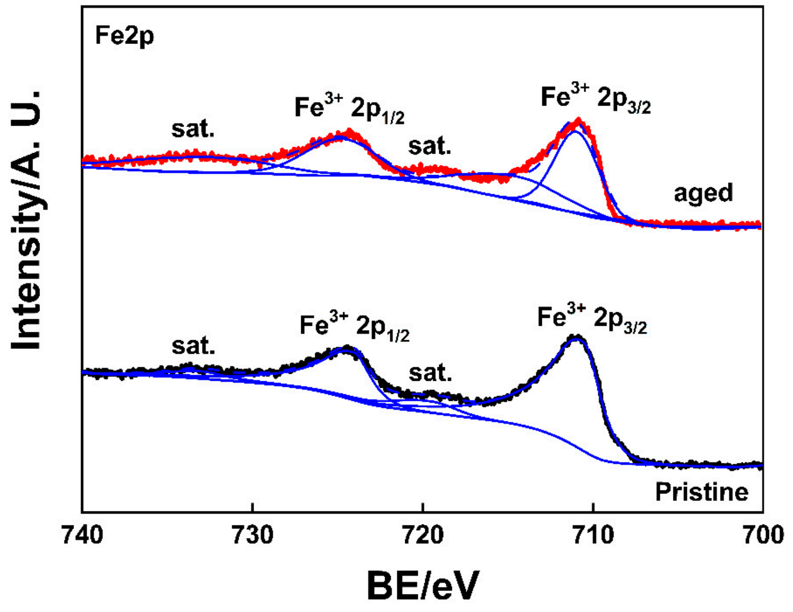

Figure S9 XPS data of Fe2p from anodic Fe foam before and after alkaline water electrolysis aging.

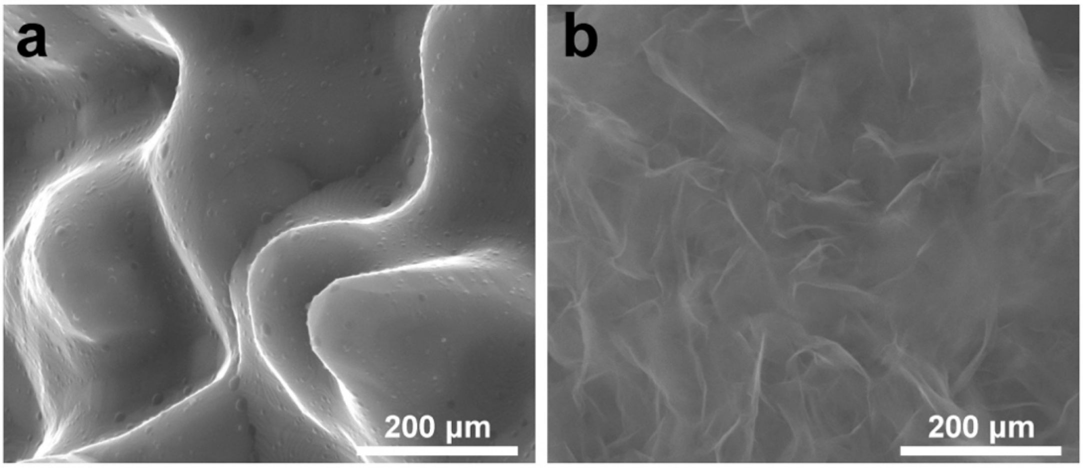

Figure S10 SEM of Ni foams before (a) and after (b) water electrolysis aging.

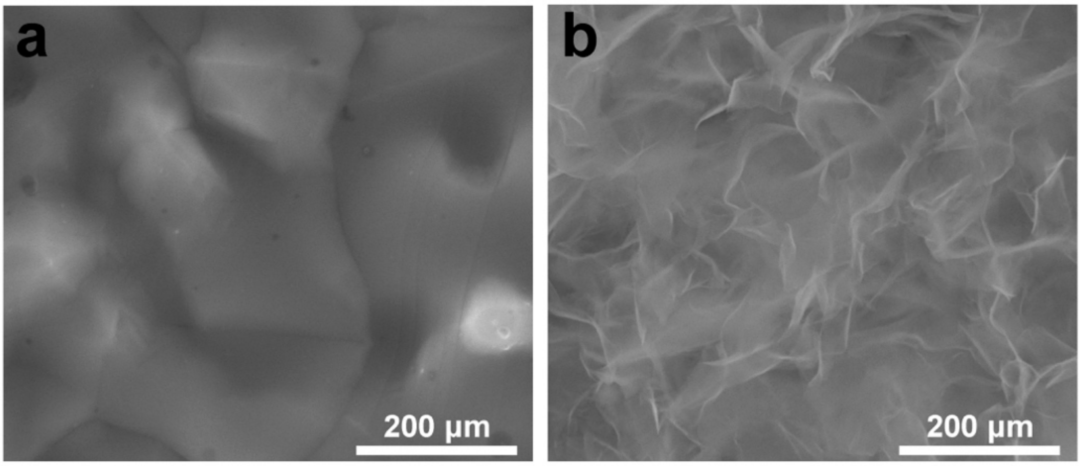

Figure S11 SEM of NiFe foams before (a) and after (b) water electrolysis aging.

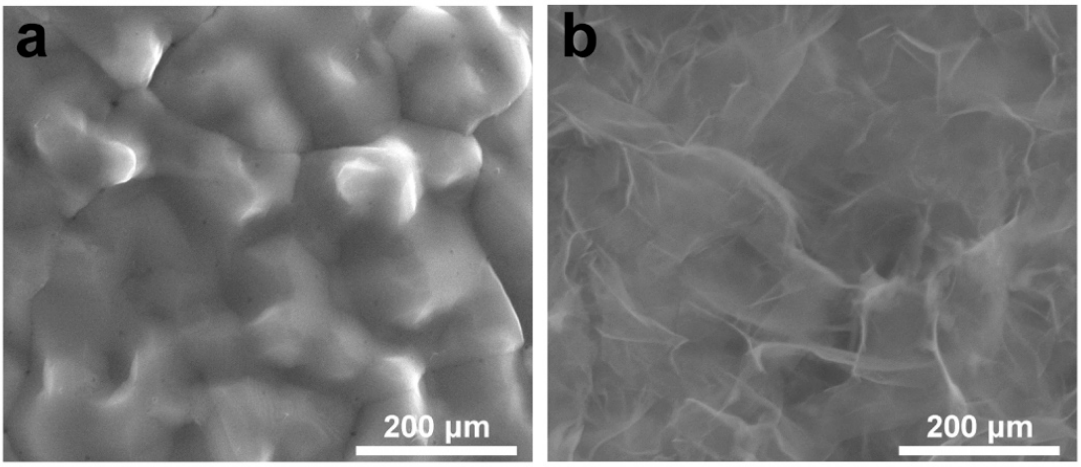

Figure S12 SEM of $NiFe_3$ foams before (a) and after (b) water electrolysis aging.

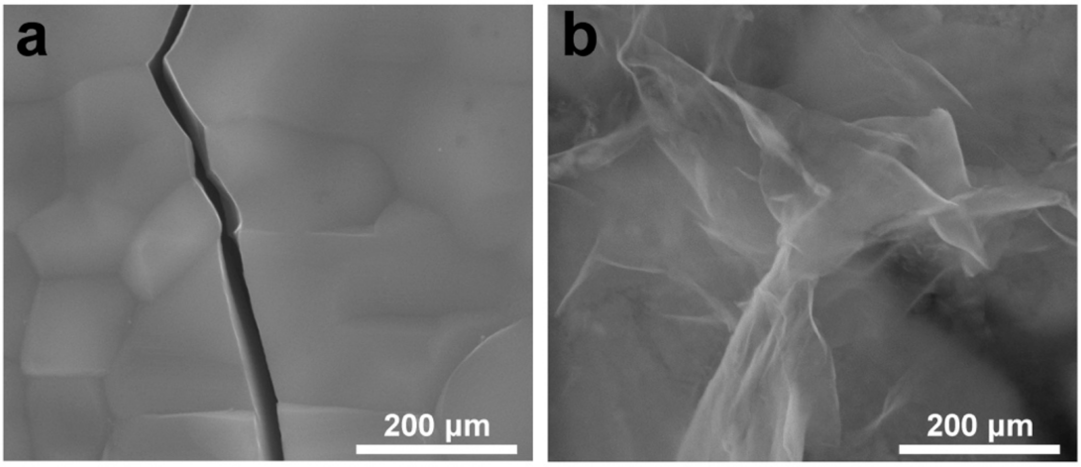

Figure S13 SEM of Fe foams before (a) and after (b) water electrolysis aging.

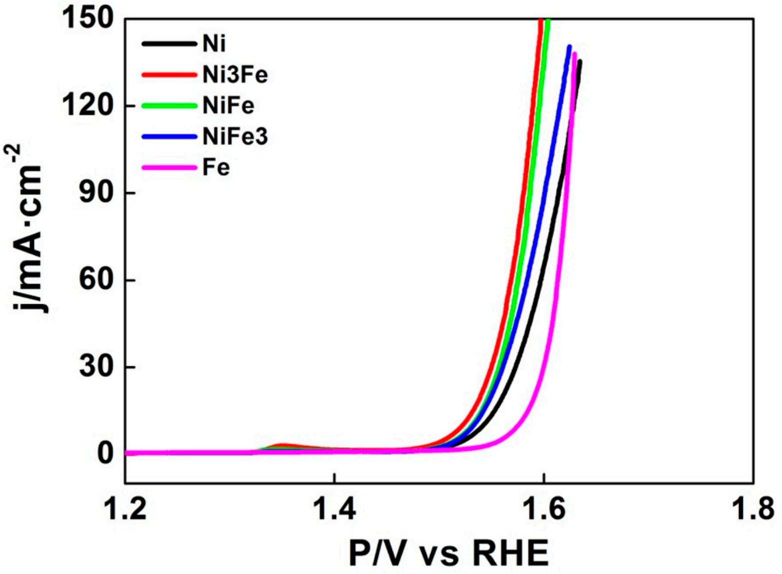

Figure S14 OER performance of pristine Ni, NiFe and Fe foams in 1 M KOH with iR compensation.

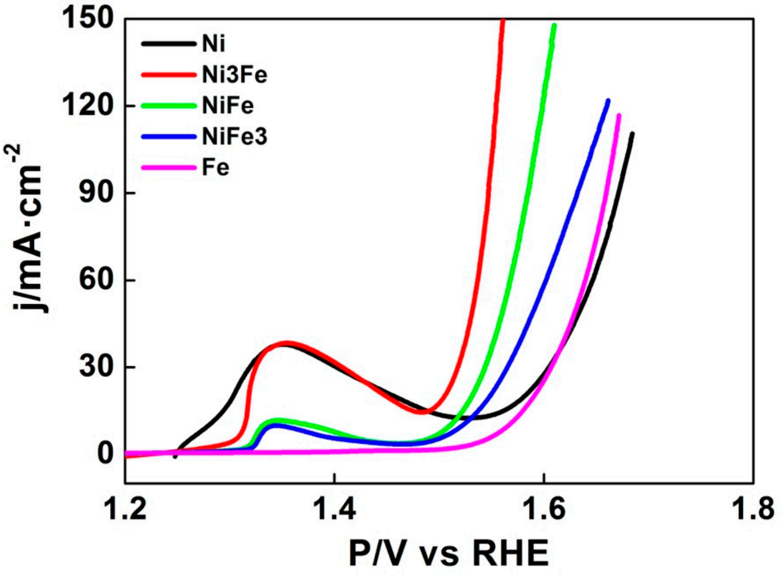

Figure S15 OER performance of aged Ni, NiFe and Fe foams in 1 M KOH with iR compensation.

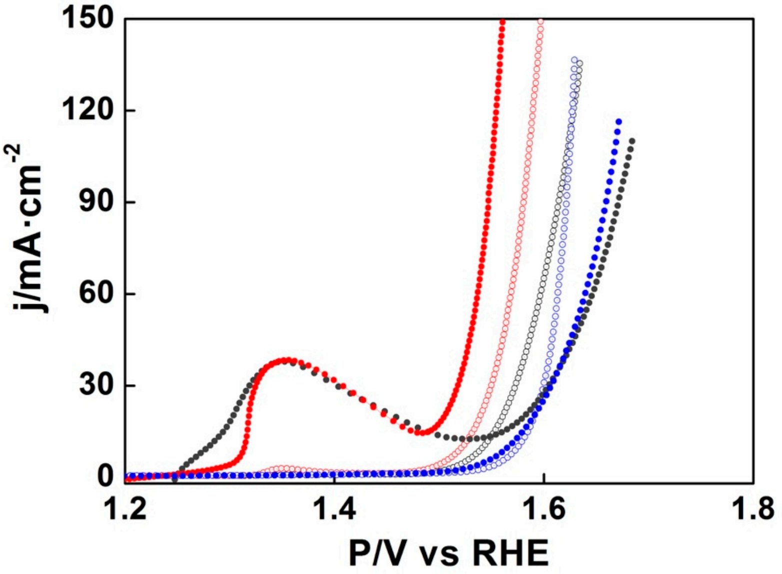

Figure S16 OER performance comparison of Ni, Ni_3Fe and Fe foams before (circle) and after (solid) electrolysis aging in 1 M KOH with iR compensation. Red dots for Ni_3Fe, blue dots for Fe and black dots for Ni.

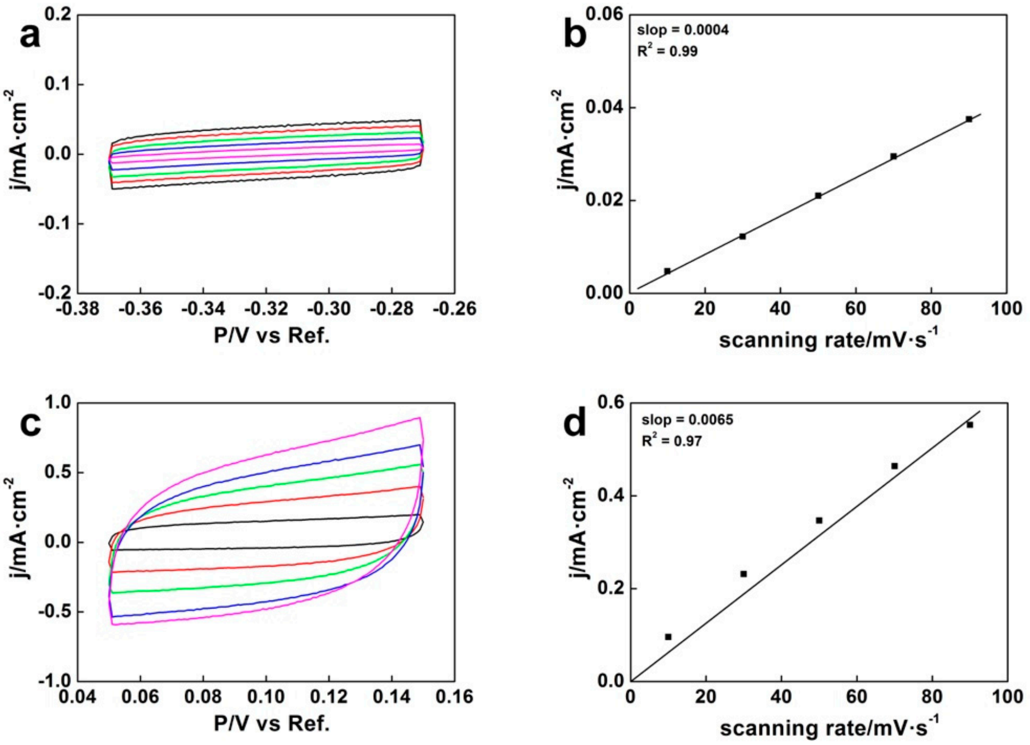

Figure S17 ECSA 100 Ni foam before (a, b) and (c, d) after electrolysis aging.

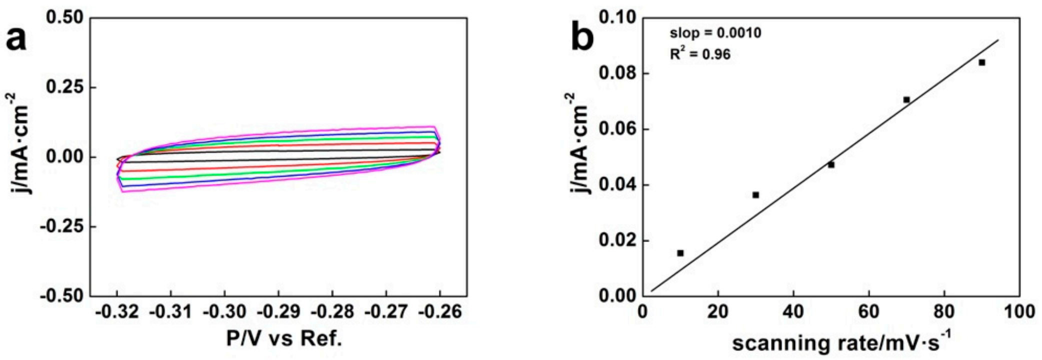

Figure S18 ECSA Ni_3Fe foam before electrolysis aging.

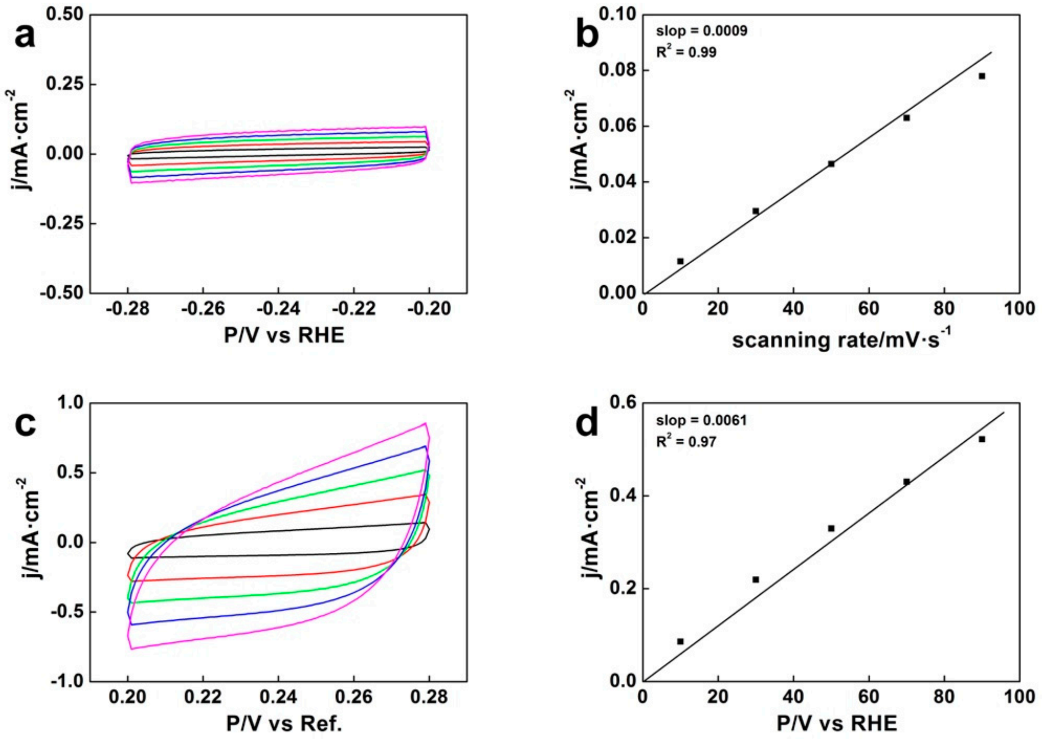

Figure S19 ECSA NiFe foam before (a, b) and after (c, d) electrolysis aging.

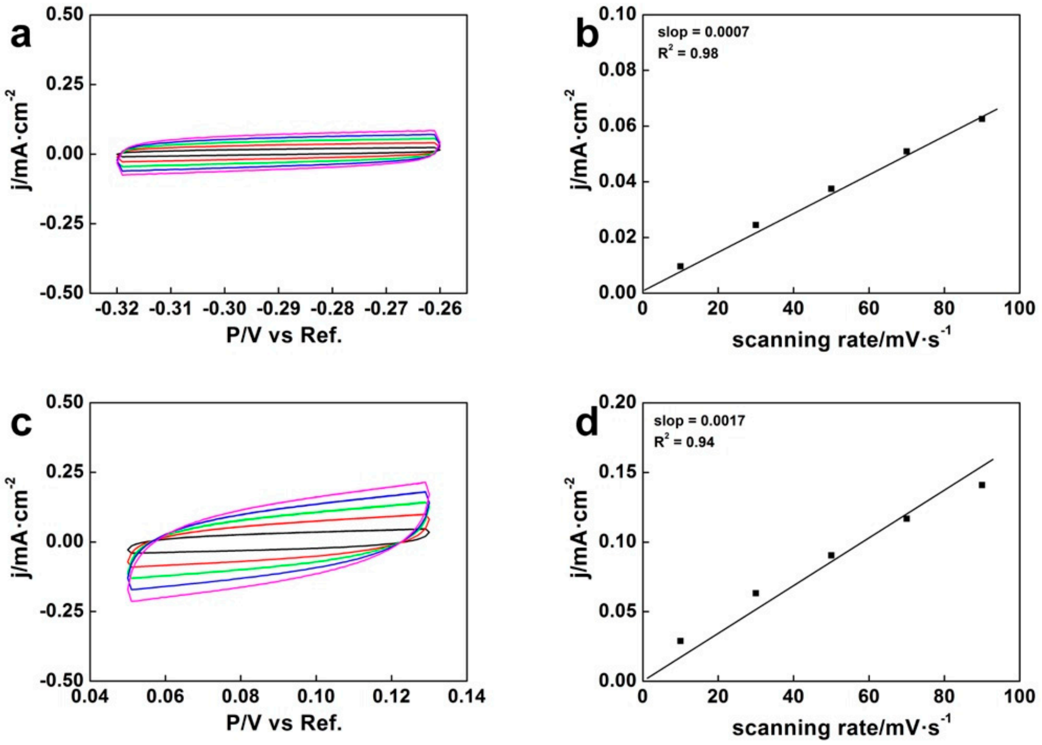

Figure S20 ECSA $NiFe_3$ foam before (a, b) and (c, d) after electrolysis aging.

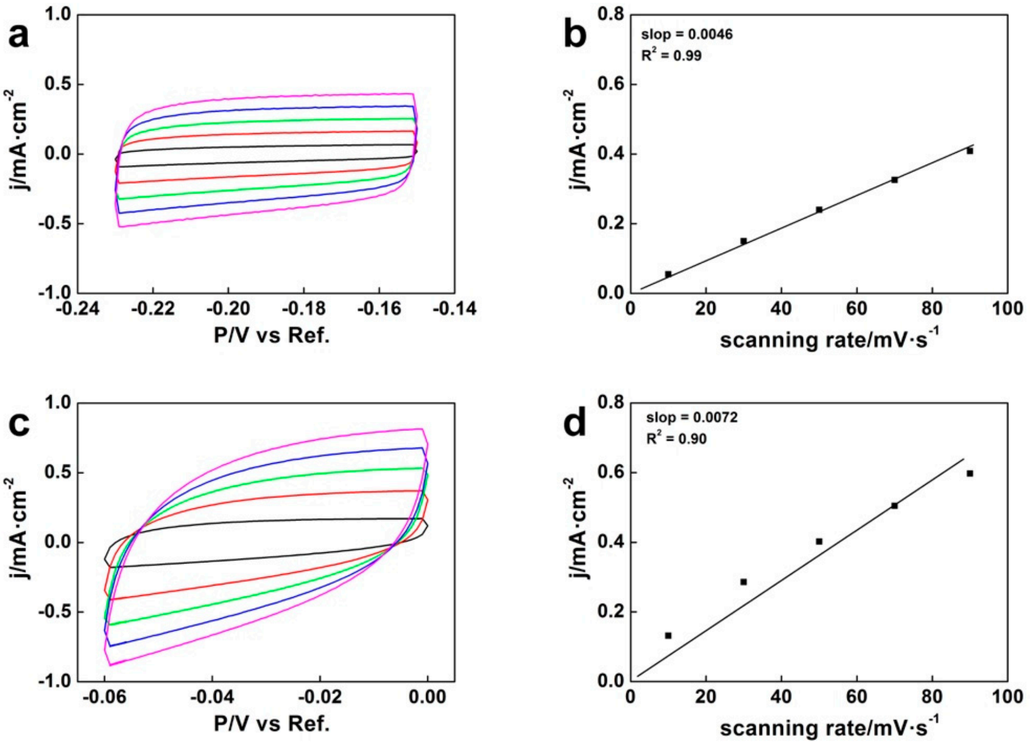

Figure S21 ECSA Fe foam before (a, b) and (c, d) after electrolysis aging.

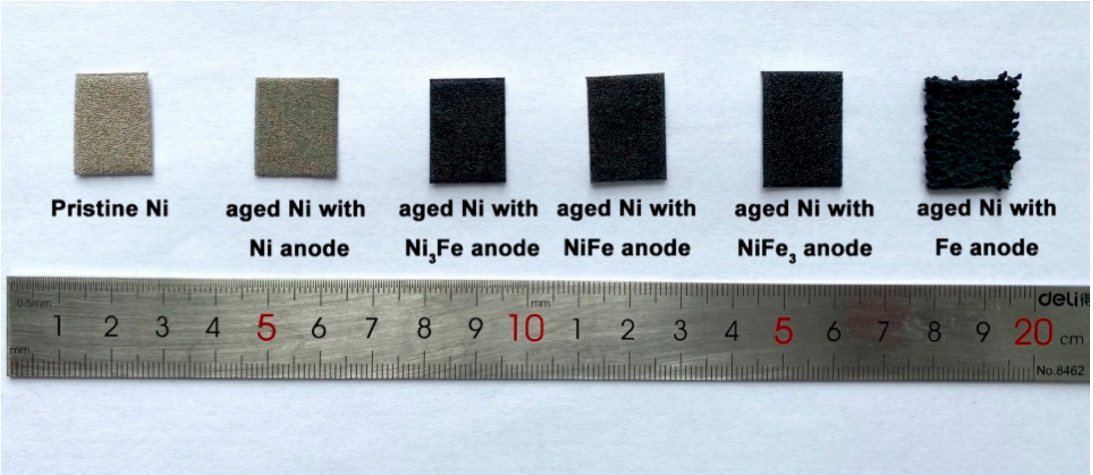

Figure S22 Photos of Ni foams before and after water electrolysis aging (wet).

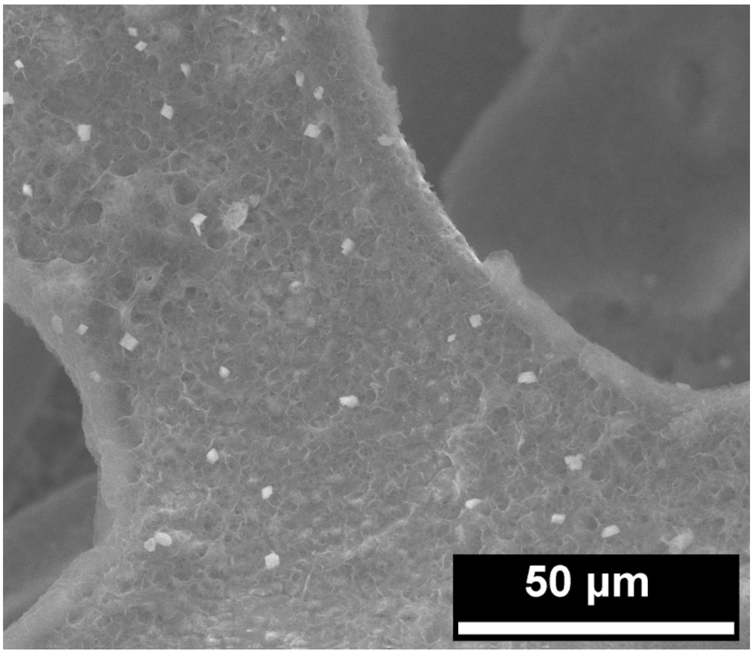

Figure S23 SEM of Ni foam cathode after water electrolysis aging with the Ni foam anode.

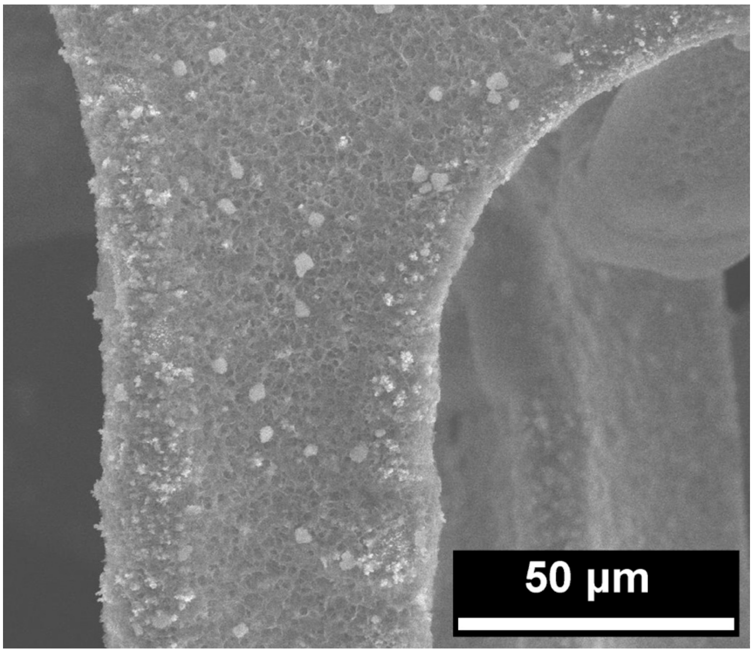

Figure S24 SEM of Ni foam cathode after water electrolysis aging with the Ni_3Fe foam anode.

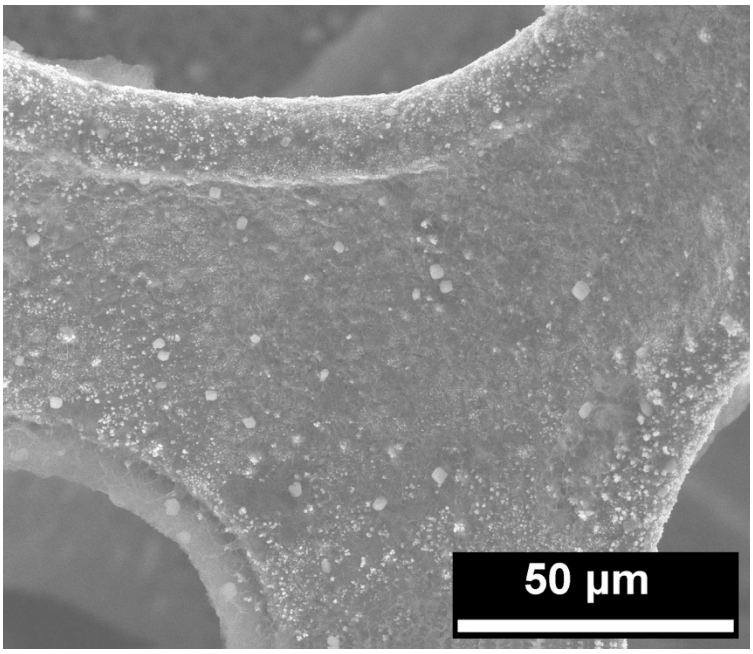

Figure S25 SEM of Ni foam cathode after water electrolysis aging with the NiFe foam anode.

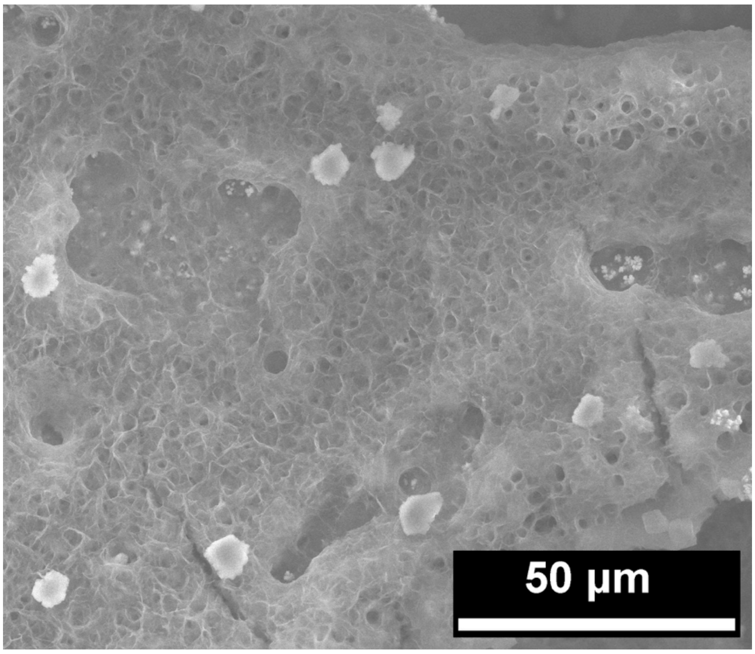

Figure S26 SEM of Ni foam cathode after water electrolysis aging with the $NiFe_3$ foam anode.

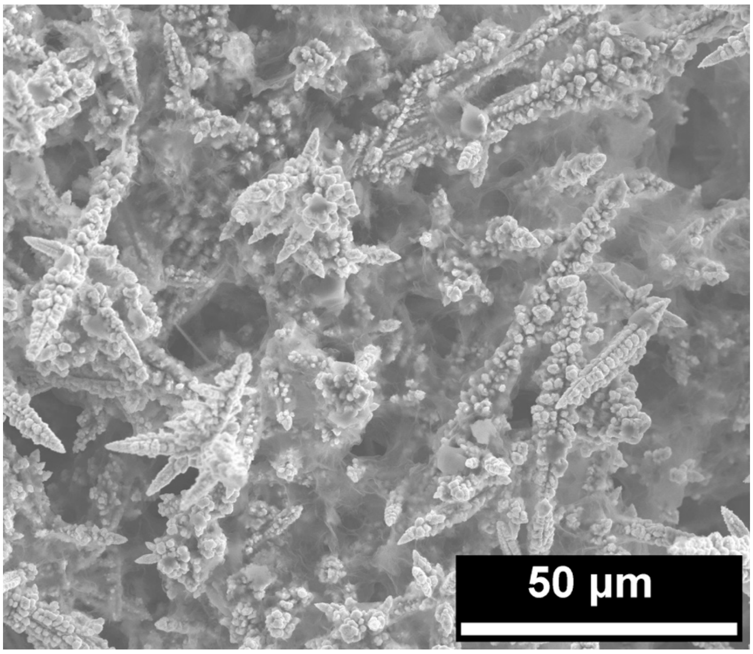

Figure S27 SEM of Fe foam cathode after water electrolysis aging with the Fe foam anode.

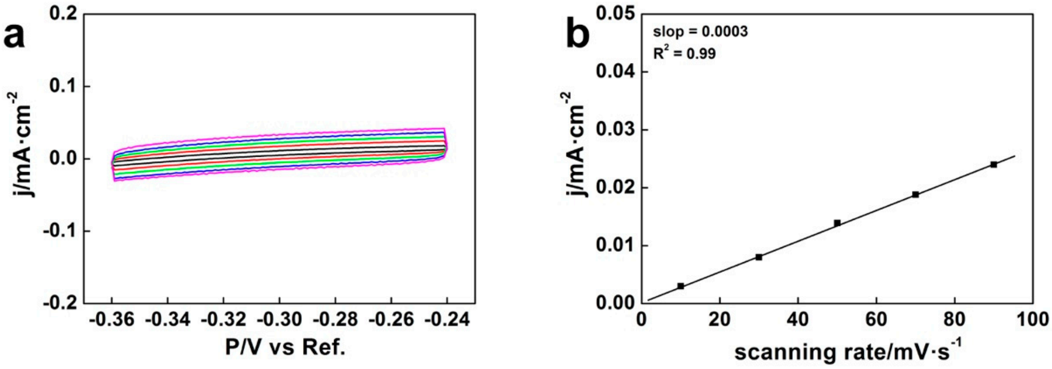

Figure S28 CV (a) and ECSA slop simulation (b) of the pristine nickel cathode.

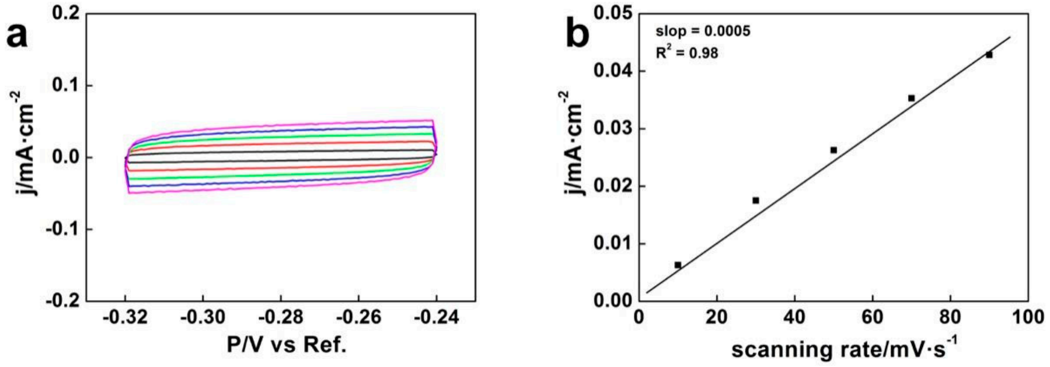

Figure S29 CV (a) and ECSA slop simulation (b) of the nickel cathode coupled with nickel anode after AWE aging.

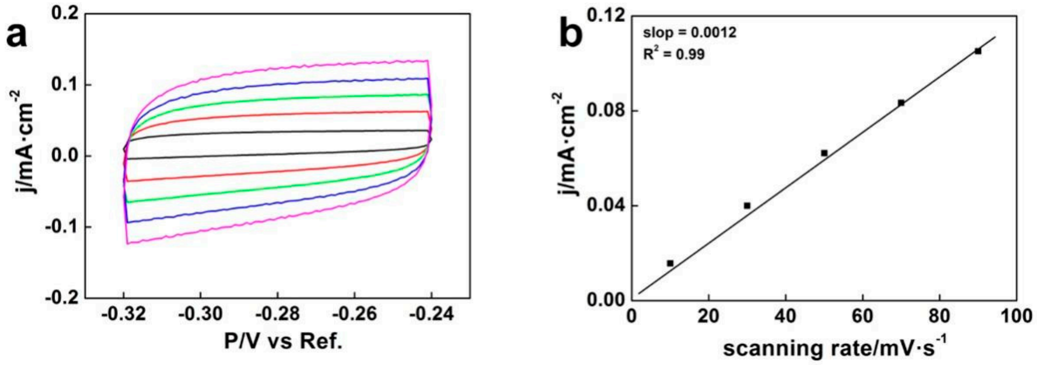

Figure S30 CV (a) and ECSA slop simulation (b) of the nickel cathode coupled with Ni_3Fe anode after AWE aging.

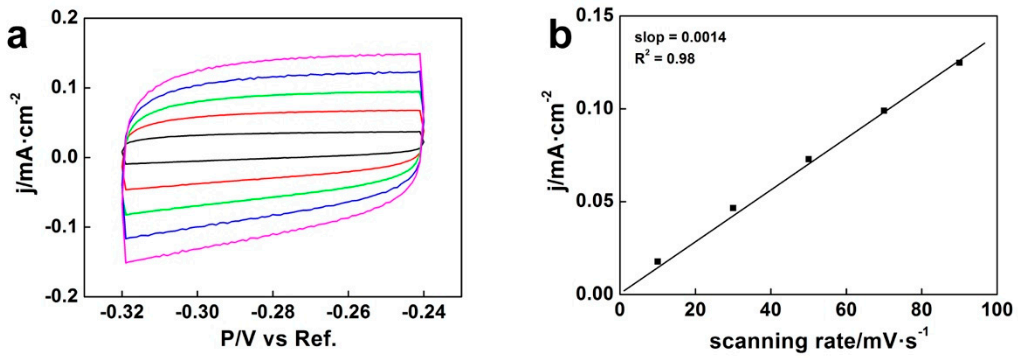

Figure S31 CV (a) and ECSA slop simulation (b) of the nickel cathode coupled with NiFe anode after AWE aging.

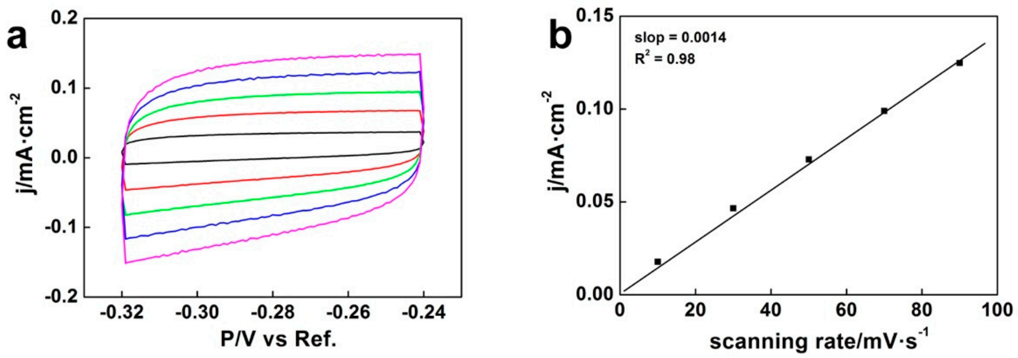

Figure S32 CV (a) and ECSA slop simulation (b) of the nickel cathode coupled with $NiFe_3$ anode after AWE aging.

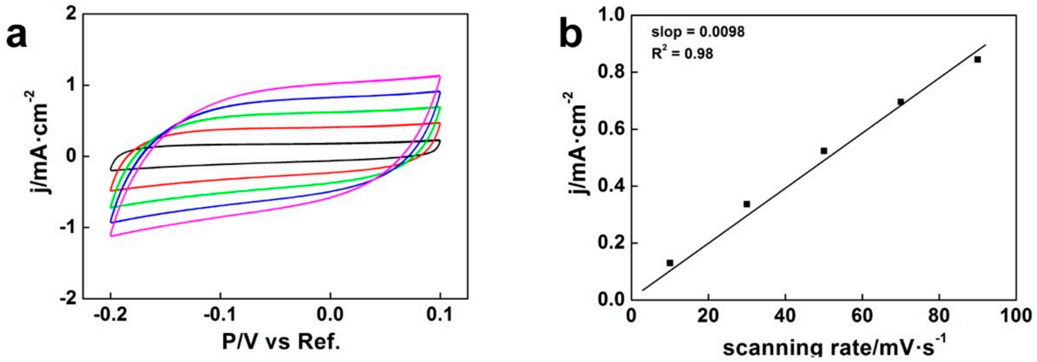

Figure S33 CV (a) and ECSA slop simulation (b) of the nickel cathode coupled with Fe anode after AWE aging.

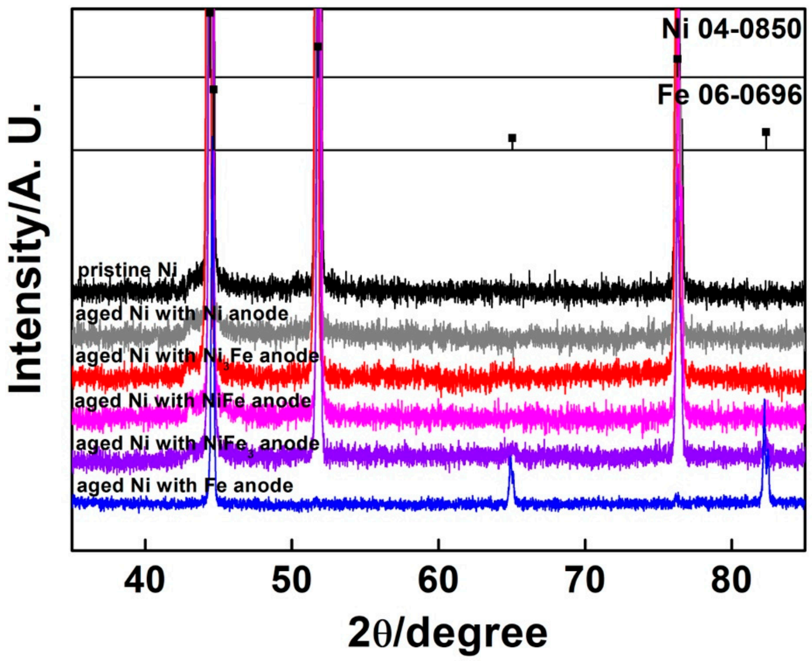

Figure S34 XRD patterns of the pristine Ni and aged Ni cathodes.

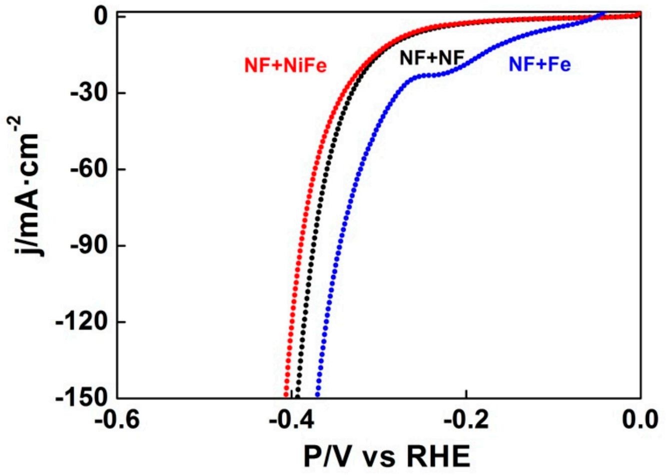

Figure S35 LSVs (a) of aged nickel foam cathodes with the couplet cathodes of Ni_3Fe, Fe and Ni foams before and after alkaline water electrolysis aging with 80% iR compensation.

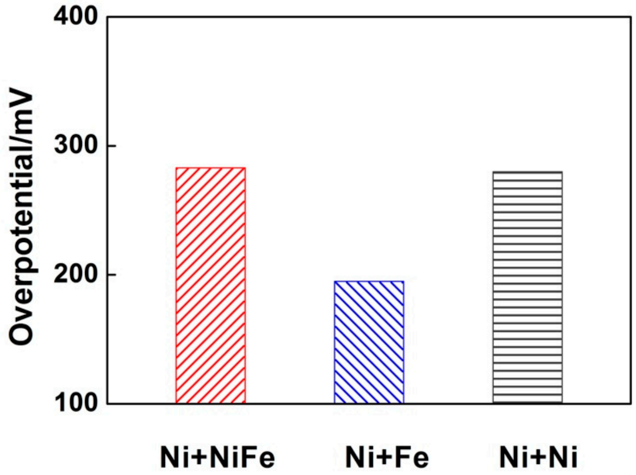

Figure S36 The corresponding overpotential at 10 mA·cm^{-2} from **Figure S35**.

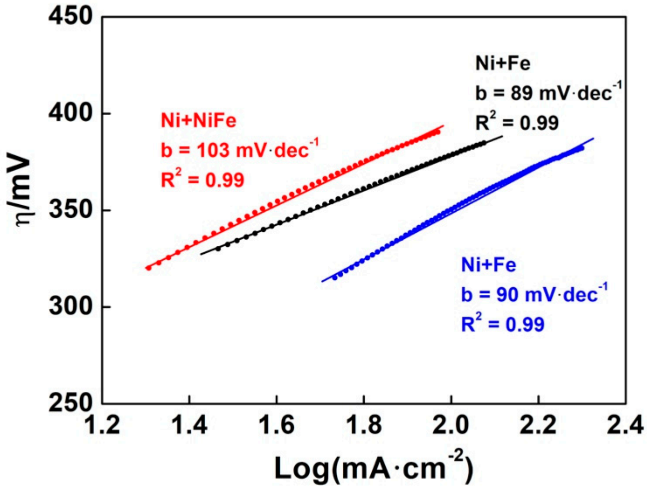

Figure S37 The derived Tafel slope values from **Figure S34**.
